# Ketogenic Interventions in Autosomal Dominant Polycystic Kidney Disease: A Comprehensive Review of Current Evidence

**DOI:** 10.3390/nu16162676

**Published:** 2024-08-13

**Authors:** Carla Pezzuoli, Giuseppe Biagini, Riccardo Magistroni

**Affiliations:** 1 Clinical and Experimental Medicine PhD Program, University of Modena and Reggio Emilia, 41125 Modena, Italy; carla.pezzuoli@unimore.it; 2 Division of Nephrology, Dialysis and Renal Transplantation, Azienda Ospedaliero-Universitaria Policlinico di Modena, 41125 Modena, Italy; 3 Department of Biomedical, Metabolic and Neural Sciences, University of Modena and Reggio Emilia, 41125 Modena, Italy; gbiagini@unimore.it; 4 Surgical, Medical and Dental Department of Morphological Sciences Related to Transplant, Oncology and Regenerative Medicine, University of Modena and Reggio Emilia, 41124 Modena, Italy

**Keywords:** ADPKD, ketogenic diet, ketosis, kidney cysts, PKD, kidney function decline

## Abstract

Autosomal Dominant Polycystic Kidney Disease (ADPKD) is a genetic disorder characterized by the development and enlargement of multiple kidney cysts, leading to progressive kidney function decline. To date, Tolvaptan, the only approved treatment for this condition, is able to slow down the loss of annual kidney function without stopping the progression of the disease. Furthermore, this therapy is approved only for patients with rapid disease progression and its compliance is problematic because of the drug’s impact on quality of life. The recent literature suggests that cystic cells are subject to several metabolic dysregulations, particularly in the glucose pathway, and mitochondrial abnormalities, leading to decreased oxidative phosphorylation and impaired fatty acid oxidation. This finding paved the way for new lines of research targeting potential therapeutic interventions for ADPKD. In particular, this review highlights the latest studies on the use of ketosis, through ketogenic dietary interventions (daily calorie restriction, intermittent fasting, time-restricted feeding, ketogenic diets, and exogenous ketosis), as a potential strategy for patients with ADPKD, and the possible involvement of microbiota in the ketogenic interventions’ effect.

## 1. Introduction

Autosomal Dominant Polycystic Kidney Disease (ADPKD) is one of the most common genetic disorders, with an estimated prevalence of 3.29–3.96/10,000 people in Europe [1,2,3]. The disease is characterized primarily by mutations in *PKD1* and *PKD2* genes, along with a few minor genes [4,5]. The *PKD1* and *PKD2* genes are crucial for maintaining normal kidney function. *PKD1* encodes the polycystin-1 protein, which is involved in cell–cell and cell–matrix interactions and plays a key role in the regulation of tubular structure and function within the kidneys [6]. *PKD2* encodes polycystin-2, a calcium-permeable ion channel that works in conjunction with polycystin-1. It helps regulate intracellular calcium levels, which are vital for various cellular processes, including cell proliferation, differentiation, and apoptosis. Mutations in these genes disrupt normal cellular signaling and function, leading to the formation of fluid-filled cysts in the kidneys and other organs [4,5], and consequently to a progressive decline in kidney function.

According to the European Renal Association (ERA) Registry Annual Report, patients with ADPKD were the youngest among primary kidney disease groups to undergo kidney replacement therapy, with 68% being younger than 64 years old. Furthermore, twice as many of these patients received pre-emptive kidney transplants compared to the other primary kidney disease groups [7]. The main manifestations of the disease are kidney function decline, hypertension, nephrolithiasis, gross hematuria, cyst infection, and acute and chronic pain attributed to kidney enlargement and episodes of lithiasis. Extra-renal manifestations of ADPKD include the development of cysts in other organs, especially in the liver parenchyma, which may cause pain, early satiety, gastro-esophageal reflux, and rarely, in the most severe cases, portal hypertension with ascites and pleural effusion [4]. To date, the only approved drug for this condition (Tolvaptan) reduces the progression of kidney damage by decreasing the loss of kidney function by about one-third compared to controls in published studies [8]. However, it does not stop the progression of the disease [9]. Furthermore, the drug is approved only in subjects with evidence of rapidly progressing disease [10]. Because of the side effects impacting quality of life, Tolvaptan is not always accepted by patients. Aside from Tolvaptan, there are no other specific treatments for the disease, except for strategies aimed at managing major complications, such as blood pressure control, kidney pain, kidney stones, and the occurrence of cerebral aneurysms.

The recent literature suggests that cystic cells are subject to metabolic dysregulation in the glucose pathway (Warburg effect) and mitochondrial abnormalities, leading to decreased oxidative phosphorylation and impaired fatty acid oxidation. This paved the way for new lines of research for potential ADPKD therapeutic targets [11,12]. In particular, in this review, we illustrate the latest studies on the use of ketosis induced by ketogenic dietary interventions (KDIs), as a possible therapeutic strategy for patients with ADPKD.

## 2. Brief Overview of Glucose Metabolism, Warburg Effect, and Ketosis

**Figure 1:**
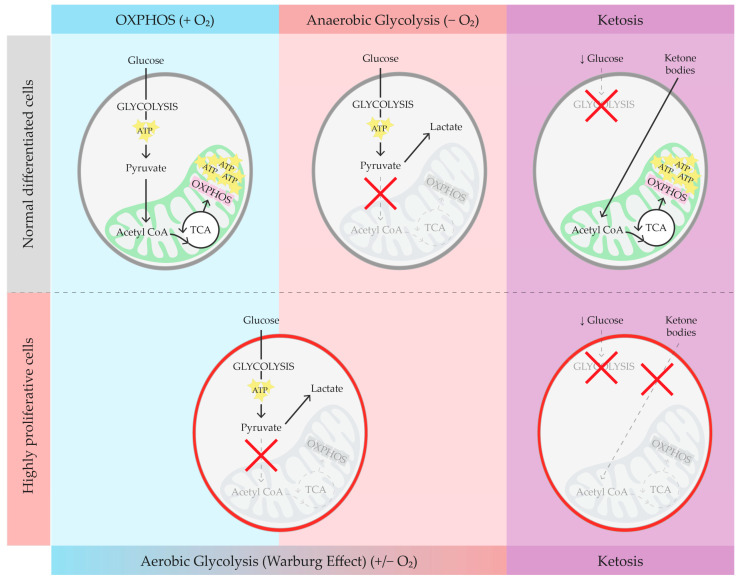
Metabolic pathways in normal and highly proliferative cells under different conditions. The image illustrates the different metabolic pathways of normal differentiated cells and highly proliferative cells under three conditions: oxidative phosphorylation—OXPHOS (in the presence of oxygen), anaerobic glycolysis (in the absence of oxygen), and ketosis. In normal cells in the presence of oxygen, glucose is metabolized through glycolysis, producing pyruvate, which enters the mitochondria to be converted into acetyl-CoA, initiating the tricarboxylic acid (TCA) cycle and ATP production via oxidative phosphorylation, yielding around 30–32 molecules of ATP per molecule of glucose. In the absence of oxygen, pyruvate is converted into lactate, yielding two molecules of ATP per molecule of glucose. During ketosis, the scarcity of glucose and the presence of ketone bodies lead to a suppression of glycolysis, with acetyl-CoA derived from ketone bodies entering the TCA cycle to produce around 20 ATP molecules. In contrast, in highly proliferative cells, even in the presence of oxygen, pyruvate is preferentially converted into lactate (aerobic glycolysis or Warburg Effect), which yields much less energy (approximately four molecules of ATP per molecule of glucose) compared to OXPHOS. Under ketosis conditions, these cells show suppression of both glycolysis and OXPHOS, highlighting the distinctive metabolic phenotype of highly proliferative cells compared to normal cells. OXPHOS: oxidative phosphorylation; ATP: adenosine triphosphate; acetyl-CoA: acetyl coenzyme A; and TCA: tricarboxylic acid cycle.

Typically, glucose is the main energy source for cells, undergoing metabolism via glycolysis (Figure 1). Facilitative transporters (GLUT1-4) usher glucose into cells, where it undergoes phosphorylation at position 6 by hexokinase enzymes (hexokinases 1 or 2, HKs). Following this, eight enzymatic reactions occur in the cytosol, resulting in the production of two pyruvate molecules and a net gain of two adenosine triphosphate (ATP) molecules for each glucose molecule. When oxygen is present, pyruvate is typically shuttled into the mitochondria for conversion into acetyl coenzyme A (acetyl-CoA), which fuels oxidative phosphorylation (OXPHOS), yielding around 30–32 molecules of ATP per molecule of glucose. However, in anaerobic conditions, pyruvate is converted to lactate in the cytosol (anaerobic glycolysis), yielding two molecules of ATP per molecule of glucose. Hyperproliferative states like cancer often favor this less-efficient metabolic process even when oxygen is available: this particular metabolic phenomenon is generally known as “aerobic glycolysis” or the “Warburg Effect” (WE). Since aerobic glycolysis yields much less energy (two molecules of ATP per molecule of glucose) compared to OXPHOS, cells compensate by upregulating glucose import and cytosolic degradation. The WE, while yielding less ATP per mole of glucose compared to OXPHOS, leaves unexploited carbonic chains available for anabolic processes, thus providing a substantial growth advantage to cancer cells [13,14]. Glucose deprivation in some cancer cell lines, called “glucose sensitive cancers” (GSCs), leads to decreased ATP production and cell death, probably triggered by a failure in ROS regulation by the antioxidant system [15]. The recent literature suggests that kidney cystic cells, similarly to GSCs, are subject to the WE (an in-depth review of these cellular mechanisms is available in more detail elsewhere [5,16]). Moreover, high urinary acetylcarnitine in a Polycystic Kidney Disease (PKD) mouse model suggests mitochondrial abnormalities, along with diminished OXPHOS and impaired fatty acid oxidation, which is one of the conditions that favor aerobic glycolysis. This has paved the way for new lines of research for potential ADPKD therapeutic targets [10,11].

A drastic reduction in circulating glucose levels occurs during a fast long enough to deplete the body’s glycogen stores. In this condition, ketone bodies (acetone, acetoacetate—AcAc, and β-hydroxybutyrate—BHB) are synthesized from the β-oxidation of fatty acids to become an alternative source of energy, generating 20 and 22.5 molecules of ATP per molecule for acetoacetate and BHB, respectively [17,18]. Blood ketone levels in healthy adult humans generally range from approximately 0.1 to 0.3 mmol/L, with maximum levels occurring after an overnight fast. These concentrations can increase significantly during metabolic stress (e.g., prolonged exercise, fasting, or pathological conditions) and can also be elevated through KDIs, achieving a state often referred to as “nutritional ketosis” (NK) [19,20,21]. While there is no official clinical definition for NK, this state is typically used as an outcome in clinical trials and is characterized by ketonemia with BHB levels of 0.5–3 mmol/L, measured in capillary blood [20,21,22].

NK can be achieved by (i) reducing the energy intake through daily calorie restriction (DCR); (ii) intermittent fasting (IMF); (iii) time-restricted feeding (TRF), a dietary approach that restricts food intake to a specific window of time each day, with fasting periods outside of that window; and (iv) ketogenic diets (KDs) (Table 1).

KDs are low-carb, high-fat diets that mimic fasting and induce ketogenesis but allow subjects to continue feeding and meeting their daily caloric needs [38]. KDs, introduced in 1920 for the treatment of drug-resistant epilepsy, are now used for a variety of other neurological [31], metabolic, and non-metabolic diseases [39], and could be potentially used in ADPKD patients to slow down the progression of the disease. There are numerous variations in KDs, often with overlapping features, and it is common for studies not to specify the exact type of KD used. This can lead to confusion and challenges in interpreting results across different studies. Currently, five main types of KD therapies are recognized: classic KD (cKD), medium-chain triglyceride diet (MCT), modified Atkins diet (MAD), low glycemic index treatment (LGIT), and the very-low-calorie KD (VLCKD) diet conceived for weight loss [31,36]. The cKD consists of a ratio of 4:1 or 3:1 g of fat to combined protein and carbohydrates. The MCT, MAD, and LGIT have been developed as normocaloric diets that allow a state of ketosis to be achieved while reducing fat intake. This modification allows for a higher protein content, necessary for growth, as well as improved tolerability and palatability [31]. The use of MAD is particularly important for the adult population, as it only requires monitoring of daily carbohydrate intake (max. 20 g/day) and a preference for lipid-rich foods, while there are no restrictions on proteins, liquids, or calories. The MAD is effective at reaching and maintaining a state of ketosis comparable to that of the cKD [34]. The International Recommendations for the Management of Adults Treated With Ketogenic Diet Therapies show that 90% of institutions use MAD, the KD variant with the highest adherence rates, due to its flexibility and ease of administration [33,35].

Finally, an alternative approach to increasing ketonemia, bypassing the metabolic shift, involves the use of supplements, which include ketogenic precursors (MCTs and 1,3-butanediol) and exogenous ketones: ketone salts (BHB or AcAc bound to sodium, potassium, calcium, or magnesium) or esters (BHB or AcAc bound to 1,3-butanediol or other alcohols), and BHB enantiomers [40,41]. Exogenous ketones can increase blood ketone levels to NK within 30 min to an hour after ingestion. However, the duration of this rise is variable, depending on the type of supplement, dosage, and individual metabolic response, often requiring multiple doses throughout the day to maintain ketosis. The need for frequent dosing is further complicated by issues such as poor palatability, high cost, and potential gastrointestinal problems [40,42]. Additionally, ketone salts may contain minerals, which can lead to elevated blood ion levels if consumed in quantities that allow the achievement of ketonemia levels comparable to those obtained through NK [43]. These supplements could be a valuable support when combined with a KD requiring high therapeutic ketosis, as is already the case with MCTs [31,33]. However, it is important to consider that exogenous ketones inhibit lipolysis, which can be counterproductive when combined with a KD aimed at reducing adipose tissue [44]. Consequently, more research is needed to fully understand the long-term benefits and potential drawbacks of using these supplements.

## 3. Ketosis in ADPKD Animal Models

In the last 10 years, several preclinical studies involving PKD animal models have been carried out to both understand the pathophysiology of the disease and identify potential new therapies [5]. In addition to studies exploring possible pharmacological interventions, considering the cysts’ defective metabolism, some authors have focused on the possibility of acting through diet manipulation (Table 2).

In 2016, Warner et al. [45] induced a 40% DCR for 6 months compared to a standard ad libitum (AL) diet in an ADPKD mouse model. After the treatment period, the nearly complete inhibition of cyst development was observed due to DCR. The authors also found that kidney inflammation decreased to the level of normal wild-type control kidneys. In addition, further to histological analysis and evaluation of acute and chronic kidney markers of integrity, a decrease in fibrosis and kidney injury was observed in DCR compared to AL. Finally, DCR mice showed a decrease in both cellular proliferation and apoptosis markers. The reduction in cystogenesis was found even with a mild DCR (10 and 20%); this result was confirmed in the study by Kipp et al. [46] on ADPKD mice fed on average 77% of the food consumed by the AL controls. The same group then confirmed in animal models that the inhibitory effects on ADPKD progression, obtained through DCR, were caused by the induction of ketosis, which impacted the cystic cells because they are metabolically inflexible and thus unable to adapt to alternative fuel sources. The researchers measured BHB levels in the ADPKD rats used for the DCR study and found significantly higher values compared to those in the control group, suggesting that mice underwent IMF leading to ketosis. To test this hypothesis, the group placed PKD rats on a TRF regime wherein they had access to food for an 8 h period [47]. After five weeks, animals on the TRF diet showed significantly less kidney cystic disease progression than those on the AL diet. TRF-treated animals had fewer and smaller cysts, improved kidney function, and increased levels of BHB, indicating induced ketosis. The research team then explored the use of a KD by giving PKD mice ad libitum access to a high-fat, very-low-carbohydrate KD. In juvenile rats treated with KD, the progression of kidney cystic disease was significantly inhibited compared to those on AL. Additionally, KD feeding led to improved kidney function and inhibited both cyst formation and expansion. In adult specimens, the overall number of cysts per animal remained unchanged with KD; nonetheless, there was a significant decrease in cyst size, suggesting that KD feeding prevents cyst enlargement in animals of that age.

Torres et al. [47] subsequently subjected Persian PKD cats to a 72 h fast, resulting in an average reduction of total kidney volume (TKV) by 15%, suggesting fluid drainage during fasting. Considering that KD increases ketonemia, to conclude the authors attempted to administer a BHB sodium/potassium salt formula to mice fed AL with normal chow. After 5 weeks, the kidneys of treated PKD animals resembled those of wild-type animals in appearance and structure. BHB-treated PKD rats showed notable reductions in kidney size and cystic area compared to controls. BHB treatment also led to improved kidney function and reduced fibrosis, myofibroblast presence, and cell proliferation. The research group was particularly interested in understanding whether ketosis could lead to inhibition of mTOR, a nutrient availability-dependent kinase involved in cellular metabolism, found to be highly activated in ADPKD [5,48]. Evidence shows that mTOR inhibitors can effectively reduce cyst growth and preserve kidney function in ADPKD animal models of ADPKD [49]. However, despite these promising preclinical results, the use of mTOR inhibitors in ADPKD patients has not been successful to date [50]. Torres et al. found that BHB inhibits mTOR in their models, and this inhibition may have contributed to the reduction in cyst growth and kidney volume [47]. Further investigation is required to understand the role of ketosis in PKD, as BHBs are important metabolic and signaling mediators [51]. Recently, Weimbs and collaborators found that the simultaneous administration of BHB sodium/potassium salt and citrate not only significantly decelerated the progression of PKD in juvenile rats, but also partially reversed established cystic disease in adult rats [52].

Hopp et al. [53] conducted a study to compare DCR, IMF, and TRF in PKD mice. In their study, only the DCR regimen proved effective in slowing the progression of cystic kidney disease. However, contrary to expectations, the lack of benefit observed with TRF does not align with Torres et al.’s findings. This discrepancy could be attributed to differences in the PKD gene variant, disease progression timing, species background, or intervention duration. Moreover, the absence of a therapeutic effect in animals on IMF may be attributed to compensatory overeating on non-fasting days. In this study, the authors observed that the therapeutic effect was evident solely in animals who followed a dietary regimen leading to weight loss. This presents another inconsistency compared to the studies by Torres and Kipp, where DCR and TRF achieved effectiveness in slowing PKD progression without a significant change in body weight (BW) compared to the control group. Once more, the choice of model or the timing of disease progression might elucidate the discordant outcomes [53].

**Table 2:** Comprehensive summary of studies on the effects of different dietary interventions on animal models of Polycystic Kidney Disease (PKD).

Intervention and Study Design		Model (Orthologous: ✓/✕)	Control Group	Kidney Outcomes	Notes
Fasting	48 h WF [47]	✕ Han:SPRD-*Cy/+* rats aged 8 wk	48 h WF WT	KHW ↓ * (male). CI ↓ *. Cysts apoptosis.	NK achieved. ↓ BW (both groups).
	24 h WF [47]	✓ *Pkd1*^cond/cond^:Nes^Cre^ mice aged 8 wk	24 h WF WT	No TKV changes. Cysts apoptosis.	NK achieved.
	72 h WF [47]	✓ *Pkd1* Persian cat aged 34.2–64.9 mo	N/A	TKV ↓ (~15%)	No NK evaluation. ↓ BW.
DCR	6 mo DCR (−40%) [45]	✓ *Pkd1*^RC/RC^ miceaged 6 wk	AL model AL WT	KHW, CI, IF, cystogenesis ↓ *.	No NK evaluation. ↓ * BW.
	3 mo DCR (−40%) [45]	✓ *Pkd1*^RC/RC^ miceaged 5.5 mo	AL model AL WT	KHW ↓ *. Improvement in BUN and CysC.	No NK evaluation.
	2 mo DCR (−10%, −20%, −40%) [45]	✓ *Pkd1*^RC/RC^ miceaged 5.5 mo	AL model	KHW ↓ *. CI ↓ (↓ * 20%, 40%).	No NK evaluation. ↓ BW.
	2 mo DCR (−40%) [45]	✓ *Pkd2*^WS25/-^ mice aged 8 wk	AL model	KHW, CI ↓ *	No NK evaluation.
	12 wk DCR (−23%) [46]	✓ *Pkd1*^cond/cond^:Nes^Cre^ mice aged 35 days	AL model DCR WT	KBW ↓ *. IF ↓	No NK evaluation.
TRF	5 wk TRF (8 h window) [47]	✕ Han:SPRD-*Cy/+* rats aged 3–8 wk	AL NC model TRF WT AL NC WT	KBW, CI, cystogenesis ↓ *. IF, SC ↓.	NK achieved.
DCR IMF TRF	3 mo DCR (−40%), IMF (−80% 3 days/wk), TRF (8 h window) [53]	✓ *Pkd1*^RC/RC^ miceaged 3 mo	AL model	KBW, CI ↓ * (DCR).	↓ * BW (DCR).
KD	5 wk AL cKD [47]	✕ Han:SPRD-*Cy/+* rats aged 3–8 wk	AL NC model AL KD WT AL NC WT	KBW, KHW, CI, cystogenesis, SC ↓ *. IF ↓.	NK achieved. ↓ * BW gain.
	4 wk AL cKD [47]	✕ Han:SPRD-*Cy/+* rats aged 8–12 wk	AL NC model AL KD WT AL NC WT	TKV, KBW, CI ↓. IF ↓ (male).	NK achieved. ↓ BW gain.
Exogenous ketosis	5 wk Na and K BHB salt in water [47]	✕ Han:SPRD-*Cy/+* rats aged 3–8 wk	Water model Water + Na/KCl model	KBW, CI, IF ↓ *. IF ↓ *. SC, cystogenesis ↓.	No BW changes.
	5 wk Na and K BHB salt and/or citrate [52]	✕ Han:SPRD-*Cy/+* rats aged 3–8 wk	Water model Analogous titrations WT	KBW, SC ↓ * (BHB, BHB/citrate). CI ↓ * (80, 160 Mm BHB; 40/60, 80/60 Mm BB/citrate).	
	5 wk Na and K BHB salt and citrate [52]	✕ Han:SPRD-*Cy/+* rats aged 8–12 wk	Water + Na/KCl model	KBW, CI ↓. SC ↓ *.	

AL: ad libitum; BHB: β-hydroxybutyrate; BW: body weight; CI: cyst index; CysC: cystatin C; DCR: daily caloric restriction; h: hours; IF: interstitial fibrosis; IMF: intermittent fasting; K: potassium; KBW: 2-kidney weight to body weight ratio; KHW: kidneys/hearth weight; KD: ketogenic diet; mo: months; Na: sodium; Na/KCl: sodium and potassium chloride; NC: normal chow; SC: serum creatinine; TKV: total kidney volume; TRF: time-restricted feeding; WF: water fasting; wk: weeks; WT: wild-type; ↓: decrease, and * for significance.

## 4. Ketosis in ADPKD Patients

There are only a few published clinical studies on the use of KDIs in ADPKD. Of these studies, some have primarily assessed the feasibility and safety of the therapy [53,54], while others have evaluated its impact in small groups of patients [55,56,57,58]. To the best of our knowledge, two RCT studies are ongoing: one on DCR and the other on MAD [59,60]. Moreover, two interventional studies with no control group are not yet recruiting: one on KD and the other on exogenous ketosis [61,62].

The first study evaluating the tolerability and safety of ketosis in ADPKD patients was the GREASE single-arm interventional pilot study [54]. Three patients with ADPKD were enrolled to follow a MAD for three months. Tolerability was assessed by a 29-item questionnaire on satisfaction, compliance, and wellness, which reported overall positive reviews. Patients reported no difficulties with food quality or quantity, nor did they find their taste challenged or experience trouble in purchasing or preparing it. The greatest difficulty regarding compliance was reported by a patient who mentioned difficulties related to the social implications of a restrictive diet and complained about the discomfort of monitoring glycemia and ketonemia. Finally, it is worth noting that the wellness area received the highest overall satisfaction scores in the questionnaire. The MAD had few predictable side effects (keto flu), mostly occurring during the initial phase, such as fatigue, muscle cramps, and constipation. Patients in the study experienced an increase in total cholesterol over the follow-up period and a decrease in blood pressure values, in line with the existing literature.

Following this first trial, Strubl et al. conducted a retrospective study to assess the safety, feasibility, and effects of KDIs in ADPKD patients. Through questionnaires, they gathered information from ADPKD patients who had previously undergone KDs and TRDs for at least 6 months [63]. The majority of participants reported an overall enhancement in their quality of life and well-being, including a reduction in disease-related pain (such as hip and/or back pain, abdominal swelling, and early satiety). Weight loss was reported by approximately 90% of the participants, a noteworthy result since adiposity, particularly visceral adiposity, is independently linked to accelerated kidney growth and reduces the effectiveness of Tolvaptan [64,65]. More than half of the patients reported improved blood pressure control, and approximately 15% reported reducing antihypertensive medication. Some participants experienced improvements in estimated glomerular filtration rate (eGFR); however, reliability was limited due to single, uncontrolled values and eGFR fluctuations. A slight increase might imply stabilized kidney function, but glomerular hyperfiltration due to increased protein intake, which could adversely affect kidney health, cannot be ruled out [63,66]. As already emphasized, a well-designed KD includes an adequate content of protein [38]. However, we know very little about the actual composition of the diets followed by the interviewed patients and their adherence to these protocols [67]. Most participants found KDIs manageable, with minimal impact on food preparation time. The majority described the diet as easy to follow and would recommend it. While half adhered daily, over 40% skipped occasionally due to practical difficulties, particularly with the KD. Despite challenges, KDIs appeared feasible for PKD patients, with manageable implementation and adherence. The side effects reported in the data collection were consistent with those found in the GREASE study. These preliminary evaluations demonstrated that KDIs are safe and feasible in ADPKD, clearing the path for controlled studies with larger patient numbers and longer duration. On safety, it is necessary to emphasize that these nutritional protocols entail significant metabolic changes and are not balanced; therefore, they require formulation by an experienced dietitian. Supplementing the KD with vitamins, minerals, and, if necessary, fiber prevents nutritional deficiencies and intestinal issues. A common misconception is that these diets must be high in protein, while a well-formulated KD is characterized by a high-fat and low-carbohydrate intake with protein content ranging from normal to moderate [38]. Moreover, the use of a diet with moderate protein content is not currently contraindicated in ADPKD patients. The latest guidelines for the nutritional treatment of ADPKD patients with chronic kidney disease (CKD) suggest considering personalized nutritional plans for these patients, as studies suggest that they may not benefit from protein restriction [68].

One potential side effect of long-term KDs may be an increased risk of developing kidney stones [69]. Uric acid stones are the most frequent type observed in KD patients, followed by calcium-based and mixed uric acid–calcium stones. The exact mechanism behind kidney stone formation on this diet remains uncertain but it could be associated with conditions like hypocitraturia and acidosis, which are typical in individuals adhering to a high-protein, low-alkali diet. Consequently, urine alkalization with oral potassium citrate, a strategy already employed in patients with drug-resistant epilepsy using KDs, should be considered in patients with ADPKD to prevent uric acid precipitation [69]. Lastly, another concern regarding the KD is its impact on cardiovascular disease (CVD) risk. To date, the long-term effects of the ketogenic diet on dyslipidemia, hypertension, and overall cardiovascular risk are still uncertain, with conflicting findings in the research [70,71,72,73]. This lack of consensus depends on several factors: heterogeneity across studies, a small number of studies in certain populations (e.g., normal-weight adults), KD composition and control diets, KD fat sources, patient adherence, and ketone concentration [70,71,74].

The 16-week duration Ren.Nu beta test program [55] adopted a plant-focused KD, with 30–40 g net carbohydrates, designed to avoid some kidney stressors (oxalate, inorganic phosphate, and purines/uric acid). The diet included a moderate intake of fish, eggs, and full-fat dairy to achieve a protein intake of ≤0.8 g/kg BW. Patients were also administered a supplement containing BHB (potassium, calcium, and magnesium BHB salts), with citrate and alkaline base, to prevent the formation of kidney stones. The study involved training patients to monitor ketosis levels alongside nutrition management. Twenty participants concluded the trial, which commenced with a one-day modified fast comprising 500–600 kcal, including moderate levels of protein and carbohydrates, followed by the actual KD. After 8 weeks of treatment, participants were advised to follow a TRF regimen for an additional 4 weeks. At the end of the program, patients reported that the program improved their PKD symptoms, in particular regarding flank pain and fatigue. The diet was appreciated and deemed feasible, except for the already familiar initial keto flu and meals eaten outside the home. Anthropometric measures, blood tests, and kidney function were analyzed post-trial, revealing weight loss, blood sugar reduction, and improvements in kidney function. However, kidney function assessment was limited to creatinine measurement and estimated glomerular filtration rate. The same authors agree that the study obviously has limitations, primarily due to the lack of a control group and the acquisition of data mainly through self-measurements and questionnaires from highly motivated individuals. Long-term, randomized clinical trials involving larger patient groups are certainly necessary to evaluate these initial findings.

## 5. Focus on RCTs in ADPKD Patients

Table 3 summarizes the completed and ongoing RCTs on KDIs in ADPKD.

In addition to their investigation on the PKD mice model, Hopp et al. carried out a parallel study on weight loss via DCR or IMF in a cohort of overweight or obese patients with ADPKD [53]. DCR involves reducing daily caloric intake without causing malnutrition, while IMF alternates periods of fasting with periods of eating (Table 1). The study was based on the premise that epidemiological observation suggests that ADPKD progression is significantly faster with increased BMI [64]. Based on previous animal studies by Warner and Torres [45,47], the authors expected a greater slowdown in the pathology in IMF patients compared to DCR ones. Surprisingly, researchers found that both groups exhibited a similar annual kidney growth rate, which was notably lower compared to historical controls. Moreover, participants who achieved clinically significant weight loss showed, on average, a cessation of kidney growth. Intriguingly, they also noticed a correlation between weight loss, decreased abdominal fat, and slower kidney growth. The findings suggest that weight loss might play a crucial role in slowing cyst growth in overweight or obese ADPKD patients. However, it is not yet possible to determine whether the decrease in BW is merely a secondary effect of the therapeutic efficacy of metabolic reprogramming achieved through dietary manipulation. Indeed, the researchers were unable to assess BHB levels and thus ketosis induction due to sample instability and limitations related to COVID-19, which hindered access to fresh samples [53]. Given these premises, the research group subsequently conducted studies on the use of TRF and DCR in overweight and obese ADPKD patients [57,60]. During these interventions, it is crucial to avoid the risk of malnutrition or dehydration, which in the long term may lead to bone loss, anemia, and other consequences. Furthermore, little is known about the long-term risks associated with these approaches. Therefore, it is advisable to regard these KDIs as formal therapies, and thus, it is necessary to provide the patient with medical follow-up and dietary support throughout the entire process.

Following a small pilot trial on short-term ketogenic intervention of KD vs. water fasting (WF) (RESET-PKD), a large RCT examining KD interventions was conducted on 63 patients [56,58]. The study included randomization into three study arms for 3 months: a KD group (<30 g/day carbohydrates and 0.8 g/kg BW/day protein), a group undergoing monthly 3-day WF, and a control group receiving routine dietary counseling for ADPKD patients. Consistent with previous studies, almost all patients in the KETO-ADPKD trial rated the KDs as feasible. The primary feasibility endpoint, which assessed both patient-reported feedback and objective evidence of ketosis (BHB ≥ 0.8 mmol/L at 75% of the in-person visits), was achieved only by the WF group, raising concerns about the feasibility of KD. However, the KD group’s failure to reach its threshold was attributed to a high predefined BHB threshold and the limited number of in-person visits while on the diet. Despite this, the KD group had significant impacts on outcomes, suggesting that lower BHB target values may still be effective. Compared to the WF and control groups, the KD group showed a potential reduction in TKV, although this change did not reach statistical significance. However, a recent post hoc analysis [75] of this study [58] suggested that examining patient subgroups based on ketone body levels reveals a significant association between higher ketone levels and greater reductions in kidney volume. Additionally, a variation in total liver volume (TLV) was noticed, although it was likely due to glycogen depletion induced by the KD. Finally, the study revealed a statistically significant decrease in body fat mass and a statistically significant improvement in kidney function (as determined by serum creatinine and cystatin C) in the KD group.

The ongoing studies have leveraged the experiences of these preliminary studies to proceed with the long-term evaluation of larger patient groups. The Nowak group’s study focuses on weight loss through DCR [60]. Investigators are conducting an RCT involving overweight or obese adults with ADPKD. Their aim is to compare the effectiveness of a weight loss intervention based on DCR to a control group in slowing kidney growth over time. Secondary outcomes include changes in abdominal fat and the effects of weight loss on biological pathways to gain mechanistic insight. Finally, GREASE2 is a 24-month RCT comparing a MAD to a balanced normocaloric diet (BND) on 92 non-obese ADPKD patients [59]. Initially, all patients will follow a BND for 1–3 months, during which they will perform weekly fasting measurements of blood glucose and BHB to establish their baseline levels. Patients randomized to the BND group will discontinue BHB measurements, while those randomized to the MAD will proceed with daily fasting blood measurements for the first month and weekly thereafter. Additionally, the MAD group will perform daily morning urinary AcAc measurements from the time of randomization. In both arms, the calorie intake will be adjusted during the study to obtain BW stability. Patients will meet the study team, composed of a nephrologist and a dietitian one month after randomization and subsequently every two months. At the 12th and 24th month, all patients will switch back to the BND before undergoing an MRI, to allow for glycogen liver replenishment. This study will assess the effect on TKV, safety, and tolerability of the MAD, and as a secondary objective, kidney function decline (based on serum creatinine and cystatin C), along with the dietary impact on the variation in exploratory prognostic biomarkers (Beta-2 Microglobulin—β2MG and Monocyte Chemoattractant Protein-1—MCP-1). Both studies will evaluate changes in TKV and abdominal adiposity through Magnetic Resonance Imaging (MRI). Moreover, GREASE2 will assess changes in TLV after glycogen replenishment.

## 6. Kidney Disease, Ketosis, and Microbiota

The interplay between kidney disease, ketosis, and gut microbiota (GM) is a rapidly evolving area of research. The kidneys and the gut microbiota engage in a complex, bidirectional relationship known as the gut–kidney axis. Recent studies have demonstrated significant alterations in the gut microbiota (GM) in kidney pathologies, characterized by the proliferation of pathogenic bacteria [76,77,78,79,80,81]. The gut–kidney axis describes a two-way interaction between the kidneys and the gut microbiota: changes in gut bacteria can affect kidney function and vice versa, contributing to systemic inflammation and disease progression [82,83,84].

An eubiotic GM includes bacteria mainly from Firmicutes, Bacteroidetes, and Actinobacteria phyla [85]. These gut bacteria produce a variety of metabolites that significantly affect kidney health. Beneficial compounds like short-chain fatty acids (SCFAs), including butyrate, acetate, and propionate, help lower colonic pH, inhibit harmful pathogens, and enhance the absorption of minerals and water, indirectly supporting kidney function. Vitamins produced by gut bacteria enhance immune function and cell health. However, the GM can be altered by factors such as uremia, dietary modifications, polypharmacy, low physical activity, constipation, and chronic inflammation. An altered GM in nephropathic patients can lead to various adverse effects, including increased intestinal permeability, systemic inflammation, and the production of uremic toxins (e.g., indoxyl sulfate, *p*-cresyl sulfate, and Trimethylamine *N*-oxide). These toxins exacerbate kidney damage, contribute to CVDs, and promote oxidative stress. Dysbiosis impairs gut barrier function, resulting in a “leaky gut” that allows bacterial translocation into the bloodstream, further aggravating inflammation and kidney disease. Chronic inflammation, driven by endotoxins and microbial metabolites, is common in CKD patients. Additionally, dietary restrictions typical in CKD, such as reduced intake of fiber-rich foods, limit essential nutrients for beneficial gut bacteria, worsening the microbiome imbalance [86]. Understanding the gut–kidney axis is crucial for developing therapeutic strategies to manage kidney diseases and improve patient outcomes.

Yacoub et al. conducted the first study on GM in PKD patients, considering different degrees of kidney failure [87]. The study strengthens the link between kidney function alterations and GM composition. Specifically, researchers observed an increase in the Lactobacillaceae family and a decrease in Oscillospira species in patients with decreased eGFR, which contrasts with previous reports showing a decrease in Lactobacillaceae and Prevotellaceae families in CKD patients. This discrepancy may be due to the confounding effects of comorbid conditions like diabetes in other studies. It also identified key metabolic pathways altered in CKD, emphasizing the role of uremic toxins produced by microbial protein catabolism, such as *p*-cresol sulfate and indoxyl sulfate. These toxins contribute to adverse cardiac outcomes and are elevated in advanced kidney disease [87]. A recent study on 25 ADPKD patients highlighted a shift towards gut dysbiosis also in PKD patients, which may influence disease progression. Healthy control subjects had a significantly higher presence of Actinobacteria, whereas ADPKD patients had increased levels of potentially harmful Enterobacteriaceae, as previously seen in chronic kidney disease patients [88,89]. PKD patients with advanced disease stages (Mayo Classes 1D and 1E) showed notably higher levels of Streptococcaceae. Early hypertension in ADPKD patients was associated with a rise in dysbiotic Proteobacteria and a reduction in probiotic Tannerelleaceae from the Bacteroidetes phylum. Elevated serum uremic toxins in ADPKD patients strongly correlated with lower eGFR and showed a positive trend with an abundance of Peptococcaceae [90].

Dietary manipulation is known to be one of the factors that can most influence the balance between bacteria [78,91,92]. Evaluating the GM in KDIs is challenging due to the limited number of studies and the significant variation in diet composition across different studies. The review by Kern et al. evaluated the impact of DCR, IMF, and TRF in vivo and in human GM [93]. Studies suggest that these KDIs may alter gut microbiota diversity, increasing beneficial bacteria, while reducing harmful ones [93,94,95]. Notably, severe DCR (60%) can cause liver inflammation and increase harmful metabolites like trimethylamine *N*-oxide (TMAO): similar microbiota changes are seen in anorexia nervosa patients and severely calorie-restricted individuals, with reduced carbohydrate-utilizing bacteria and increased mucin-degrading bacteria [93]. In KD, most studies focus on cKD in the pediatric epileptic population and VLCKD in obese patients. Both diets drastically decrease polysaccharide intake by limiting the consumption of vegetables and fruit, while other KDs allow a higher content of these two foods. Overall, these studies suggest cKD and VLCKD may harm the gut mucus barrier due to microbial changes by increasing harmful pathogens, such as *Desulfovibrio* spp. and *E. coli*, and decreasing *Bifidobacteria* [91,96,97,98,99,100]. To date, we do not have sufficient data to assess the actual changes in the gut microbiota in KDIs [32]. Large clinical randomized trials are needed to understand gut microbiota changes during different KDIs, in particular in those KDs that allow more polysaccharide intake than cKD and VLCKD.

## 7. Conclusions

The potential role of KDIs in managing ADPKD is supported by evidence from animal models and early clinical studies. These therapies, in particular KDs, appear to have a positive impact on the progression of ADPKD, reducing kidney cyst sizes and improving kidney function. However, further large-scale clinical studies are needed to confirm these preliminary findings and evaluate their long-term safety and effects on GM. Moreover, further research is necessary to determine the optimal timing and safety of KDIs for ADPKD patients at different stages of disease progression. Many studies have focused on overweight or obese patients requiring weight loss, but research is also needed in normal-weight individuals, where either maintaining weight or achieving a minimal weight loss is desirable. It is crucial to formulate KDs carefully to avoid nutritional deficiencies, reduce side effects, prevent the formation of kidney stones, and mitigate the risk of long-term cardiovascular effects. Furthermore, careful medical monitoring of patients and dietitian support to improve adherence during the implementation of these therapies is necessary.

## Figures and Tables

**Table 1:** Ketogenic dietary interventions (KDIs) achieving nutritional ketosis (NK).

KDI	Overview	Evidence	Comments
DCR [23,24,25,26]	- Energy intake reduction - Balanced macronutrients - Limited data on NK achievement	- Weight loss - Metabolic health	Challenging long-term. Unclear evidence on achieving NK.
IMF [27,28,29]	- Alternated fasting and eating periods (varied fasting schedules) - Generally low carbohydrate - NK during fasting	- Weight loss - Metabolic health	Different fasting schedules: reduced or normocaloric intake, and fasting period varying from hours (e.g., 16/8) to days (e.g., 5:2).
TRF [27,29,30]	- IMF schedule limiting daily caloric intake to a specific time window - NK during fasting hours	- Weight loss - Metabolic health	Different eating window duration.
cKD [31,32]	- Individualized calorie and protein intake - High fat (about 90%) - Low carbohydrate (<10%) - 4:1 or 3:1 ratio of fats to carbohydrates + proteins - Sustained NK	- Glut1D and PDHD treatment of choice - DRE - Some metabolic disorders	Requires the use of specifically calculated recipes measured in grams to meet the patient’s needs.
MCT [31,32,33]	- Uses MCTs to enhance ketosis - Individualized calorie and protein intake - Moderate fat (65–75%, at least 30% as MCTs) - Low carbohydrate	- DRE - Some metabolic disorders	Requires the use of specifically calculated recipes measured in grams. Greater food variety compared to cKD.
MAD [31,32,33,34,35]	- Individualized calorie and protein intake - Moderate protein intake - Low carbohydrate (15–30 g/day) - High-fat diet - Sustained NK	- DRE - Some metabolic disorders - Weight loss - Metabolic health - Some neurologic (e.g., migraine) and general medical conditions (e.g., PCOS and GSCs)	Food can be measured using standard household measurements.
LGIT [31,32,33]	- Limits carbohydrates to 40–60 g/day with GI < 50 - Focus on glycemia control rather than NK	- DRE - Weight loss - Metabolic health	Food can be measured using standard household measurements
VLCKD [36,37]	- DCR (≤800 calories/day) - Low carbohydrate (<50 g/day) - 1–1.5 g of protein/kg IBW/day - 15–30 g of fat/day - Sustained NK	- Severe obesity and/or comorbidities	Rapid weight loss effects but requires monitoring to avoid nutritional deficiencies.

cKD: classic ketogenic diet; DCR: daily caloric restriction; DRE: drug-resistant epilepsy; Glut1D: glucose transporter 1 deficiency; GI: glycemic index; GSCs: glucose sensitive cancers; IBW: ideal body weight; IMF: intermittent fasting; KDI: ketogenic dietary intervention; LGIT: low glycemic index treatment; MAD: modified Atkins diet; MCT: medium-chain triglyceride diet; NK: nutritional ketosis; PDHD: pyruvate dehydrogenase deficiency; PCOS: polycystic ovary syndrome; TRF: time-restricted feeding; and VLCKD: very-low-calorie ketogenic diet.

**Table 3:** Ketogenic diet interventions (KDIs) in ongoing and completed RCTs in Autosomal Dominant Polycystic Kidney Disease (ADPKD).

KDI	Study Design	Length and Sample	BMI	Weight Loss Outcomes	Kidney Outcomes	Status Completed: ✓, Recruiting: ●
DCR vs. IMF (both with ~34% weekly energy deficit)	2 EAs, masked (Inv, OA)	12 mo (*n* = 29)	25–45	↓ BW, SAT, VAT, TAT (MRI) (independent of KDI)	↓ htTKV correlated with BW VAT and TAT loss independent of KDI. No changes in eGFR.	✓ [53]
TRF (8 h window) vs. HE	1 EA, 1 CA, masked (Inv, OA)	12 mo (*n* = 29)	25–45	↓ BW (independent of KDI)	↓ htTKV correlated with BW and VAT loss independent of KDI.	✓ [57]
KD (carbohydrate < 30 g/day, 0.8 g/kg BW protein intake) and 3-days WF vs. AL diet	2 EAs, 1 CA, no masking	3 mo (*n* = 63)	18.6–34.9	↓ BW and significant ↓ WC (KD and WF), significant ↓ fat mass (KD), ↓ lean mass (KD and WF)	Significant creatinine-based and CysC-based eGFR ↑ with KD. Significant htTKV ↓ in a subset of KD patients reaching NK at 2/3 study visits.	✓ [58,75]
DCR (30%) and increased physical activity vs. SNC	1 EA, 1 CA, masked (Inv, OA)	24 mo (*n* = 126)	25–45	N/A	N/A	● [60]
MAD (<20 g/day of carbohydrates) vs. BND	1 EA, 1 CA, masked (Inv, OA)	24 mo (*n* = 92)	20–30	N/A	N/A	● [53]

AL: ad libitum; BMI: body mass index (kg/m^2^); BND: balanced normocaloric diet; BW: body weight; CA: control arm; CysC: cystatin C; DCR: daily caloric restriction; EA: experimental arm; eGFR: estimated glomerular filtration rate; HE: healthy eating recommendations; h: hours; htTKV: height-adjusted total kidney volume; IMF: intermittent fasting; Inv: investigator; KD: ketogenic diet; KDI: ketogenic dietary intervention; MAD: modified Atkins diet; mo: months; MRI: magnetic resonance imaging; OA: outcomes assessor; SAT: subcutaneous abdominal adipose tissue volume; SNC: single nutritional consultation; TAT: total abdominal adipose tissue volume; TRF: time-restricted feeding; VAT: visceral adipose tissue volume; WF: water fasting; ↓: decrease and ↑: increase.
